# The lateral hip instability test: Diagnostic accuracy for lateral or posterolateral femoral head undercoverage

**DOI:** 10.1002/jeo2.70296

**Published:** 2025-07-02

**Authors:** Michael Wettstein, Kinga Michalewska, Sonia Ramos‐Pascual, Stefan Hefer, Elyazid Mouhsine, Mo Saffarini

**Affiliations:** ^1^ Centre Médical ITOLS Lausanne Switzerland; ^2^ Clinique La Prairie Clarens Switzerland; ^3^ Hôpital de Rennaz HRC Rennaz Switzerland; ^4^ ReSurg SA Nyon Switzerland

**Keywords:** acetabular dysplasia, clinical test, femoral head undercoverage, hip instability, retroversion

## Abstract

**Purpose:**

To (i) describe the lateral hip instability test, developed to discriminate between stable versus unstable hips with lateral or posterolateral femoral head undercoverage, (ii) evaluate differences between painful hips that tested positive versus negative, and (iii) evaluate the accuracy of this test as defined by radiographic references for acetabular dysplasia and/or retroversion.

**Methods:**

A consecutive series of patients were evaluated for hip pain from 1 January 2019 to 31 January 2021. Routine assessment included the new lateral hip instability test, which is positive when inducing deep lateral hip pain and consists of maximum passive adduction of the painful hip, and application of a force in the long axis of the femur. Descriptive statistics were used to compare patients with positive versus negative lateral hip instability tests. The accuracy of the test was assessed using a lateral centre‐edge angle (LCEA) ≤ 20° for frank dysplasia, LCEA ≤ 25° for borderline dysplasia and a combination of positive cross‐over sign, posterior wall sign and ischial spine sign for acetabular retroversion.

**Results:**

Sixty‐nine of the 159 (43%) painful hips had a positive lateral hip instability test. Positive hips had higher Tönnis angle (9 ± 8° vs. 3 ± 5°, *p* < 0.001), lower LCEA (22 ± 8° vs. 29 ± 6°, *p* < 0.001) and greater proportions of frank dysplasia (49% vs. 6%, *p* < 0.001), borderline dysplasia (70% vs. 24%, *p* < 0.001) and acetabular retroversion (52% vs. 27%, *p* = 0.002). The lateral hip instability test had a sensitivity of 63% and a specificity of 96% for detecting borderline dysplasia and/or acetabular retroversion, a sensitivity of 68% and a specificity of 87% for detecting frank dysplasia and/or acetabular retroversion.

**Conclusions:**

The lateral hip instability test can discriminate between stable versus unstable painful hips with lateral and/or posterolateral femoral head undercoverage. When the test is positive, clinicians can be quite certain that a patient has hip instability resulting from lateral and/or posterolateral undercoverage.

**Level of Evidence:**

Level II.

**Abbreviations**
AIIS: anterior inferior iliac spine sign
COS: cross‐over sign
CT: computed tomography
FADIR: flexion adduction internal rotation
ISS: ischial spine sign
LCEA: lateral centre‐edge angle
MRA: magnetic resonance arthrogram
PWS: posterior wall sign
ROM: range of motion

## INTRODUCTION

Acetabular dysplasia and retroversion are two pathologies associated with undercoverage of the femoral head [12, 16, 22, 32, 41], which can result in hip instability. The diagnosis of both these pathologies is based on radiographic assessment, with frank dysplasia defined as lateral centre‐edge angle (LCEA) < 20°, borderline dysplasia defined as LCEA < 25° [3, 19, 44] and retroversion defined as a combination of positive cross‐over sign (COS), posterior wall sign (PWS) and ischial spine sign (ISS) [1, 38, 44].

Many types of clinical tests are used to diagnose hip instability, including established tests [6, 10, 13, 30, 33, 42, 45], such as the dial test [26], the axial distraction test [33], the posterior apprehension test [18, 36] and the Flexion Abduction External Rotation test (FABER) [29], as well as newer tests, such as the Prone Apprehension Relocation Test (PART) [39] and the Foot Progression Ankle Walking test (FPAW) [28]. However, none of these clinical tests are designed to diagnose lateral or posterolateral hip instability related to lateral or posterolateral undercoverage of the femoral head.

Hence, a new clinical test, the lateral hip instability test, has been developed by the senior surgeon (MW), to discriminate between stable versus unstable painful hips with lateral or posterolateral undercoverage of the femoral head. The purpose of the current study was to (i) describe the lateral hip instability test, (ii) evaluate clinical and radiographic differences between painful hips that tested positive versus negative, and (iii) evaluate the accuracy of the new clinical test to detect hip instability, as defined by current radiographic references for acetabular dysplasia and/or retroversion.

## METHODS

### Study design and patient selection

The authors retrospectively assessed all consecutive patients who were seen by the senior surgeon (MW) for hip pain from January 2019 to January 2021. Routine assessment included medical history, clinical examination and radiographic evaluation of both hips. Clinical examination comprised a battery of tests, including the new lateral hip instability test, which was systematically performed by the surgeon (MW) before consulting the patient's radiographs. Patients were included in the study if they were between 18 and 50 years old and had at least one native painful hip. Patients were excluded if they had (i) a history of ipsilateral acetabular or proximal femoral fracture, (ii) previous osteotomies of the pelvis or ipsilateral proximal femur, (iii) ipsilateral hip osteoarthritis (Tönnis Grade > 1), (iv) concomitant ipsilateral knee or back pathologies, (v) neuromuscular disorders or (vi) more than 6 months between acquisition of radiographs and clinical examination. This left a cohort of 140 patients (159 painful hips and 108 asymptomatic hips) (Table 1). The asymptomatic hips were used as a control group to compare against the symptomatic hips. All patients provided written informed consent for the use of their anonymised data. Ethics approval was deemed unnecessary, since the test is a part of the surgeon's standard of care and is performed and documented for every patient consulting for hip pain.

**Table 1:** Characteristics of included hips.

	Painful hips (*n* = 159)			Asymptomatic hips (*n* = 108)			*p* value
	Mean	±SD	Range	Mean	±SD	Range	
	*n*	%		*n*	%		
Patient characteristics							
Age	34.3	±8.9	18.0–49.6	35.4	±8.6	18.0–49.9	0.349
Sex: female	107	67%		72	67%		1.000
Painful hip: left	77	48%					
Duration of hip pain (months)	31.2	±38.9	0.5–240.0				
Hip pain while sitting	101	64%					
Hip pain while walking	134	84%					
ROM (°)							
Flexion	101.6	±5.6	90.0–120.0	104.6	± 5.2	95.0–120.0	**<0.001**
Extension	9.9	±1.0	0.0–10.0	10.0	± 0.5	5.0–10.0	0.527
External rotation	38.5	±10.4	15.0–70.0	37.0	± 9.7	20.0–70.0	0.316
Internal rotation	15.8	±13.5	−15.0^a^ to 60.0	20.7	± 12.6	−10.0^b^ to 70.0	**<0.001**
Abduction	44.3	±7.8	20.0–60.0	44.7	± 7.3	25.0–70.0	0.868
Adduction	29.8	±1.2	20.0–30.0	30.0	± 0.0	30.0–30.0	0.098
Clinical examination							
Painful gluteal muscles	19	12%		1	1%		**0.001**
Painful fascia lata	16	10%		0	0%		**<0.001**
Positive FADIR	140	88%		11	10%		**<0.001**
Positive apprehension test	46	29%		0	0%		**<0.001**
Positive lateral hip instability test	69	43%		4	4%		**<0.001**
Radiographic evaluation							
Tönnis grade							
0	121	76%		94	87%		**0.028**
1	38	24%		14	13%		
Tönnis angle (°)	5.6	±7.0	−5.0 to 40.0	4.9	±6.0	−5.0 to 21.0	0.568
LCEA (°)	26.1	±7.6	0.0–42.0	27.0	±7.2	7.0–45.0	0.449
LCEA ≤ 20°	39	25%		18	17%		0.131
LCEA ≤ 25°	70	44%		43	40%		0.529
Positive COS	128	81%		79	73%		0.180
Positive PWS	82	52%		49	45%		0.383
Positive ISS	64	40%		37	34%		0.369
Positive: COS, PWS, ISS	60	38%		35	32%		0.435
Positive AIIS	109	69%		62	57%		0.070

*Note*: Bold values indicate a statistically significant difference.

Abbreviations: AIIS, anterior inferior iliac spine sign; COS, cross‐over sign; FADIR, Flexion Adduction Internal Rotation; ISS, ischial spine sign; LCEA, lateral centre‐edge angle; n, number; PWS, posterior wall sign; ROM, range of motion; SD, standard deviation.

^a^ One hip was blocked at 15° of external rotation (no internal rotation possible).

^b^ The contralateral hip was blocked at 10° of external rotation (no internal rotation possible).

The following data were extracted from the medical records:

– Patient characteristics: age, sex, painful side, duration of hip pain, presence of hip pain while sitting and walking;
– Clinical examination of the painful hip including range of motion (ROM), testing of hip muscles, Flexion Adduction Internal Rotation (FADIR) test (also known as impingement test) [9, 15], anterior apprehension test (also known as HyperExtension External Rotation test [HEER]) [10, 13, 18, 30] and lateral hip instability test;
– Radiographic parameters measured on standardised anteroposterior pelvic radiographs [37, 40, 43]: Tönnis grade, Tönnis angle (also known as acetabular index angle or acetabular roof angle), LCEA, COS, PWS, ISS and anterior inferior iliac spine sign.

As part of routine preoperative assessment, patients who required and consented to surgery had a hip magnetic resonance arthrogram (MRA). For this subgroup, the presence of labral hypertrophy and its location on MRA images were reported. Labral hypertrophy is considered a sign of hip instability [2, 6, 15, 23, 24, 25, 34].

### Lateral hip instability test

**Figure 1:**
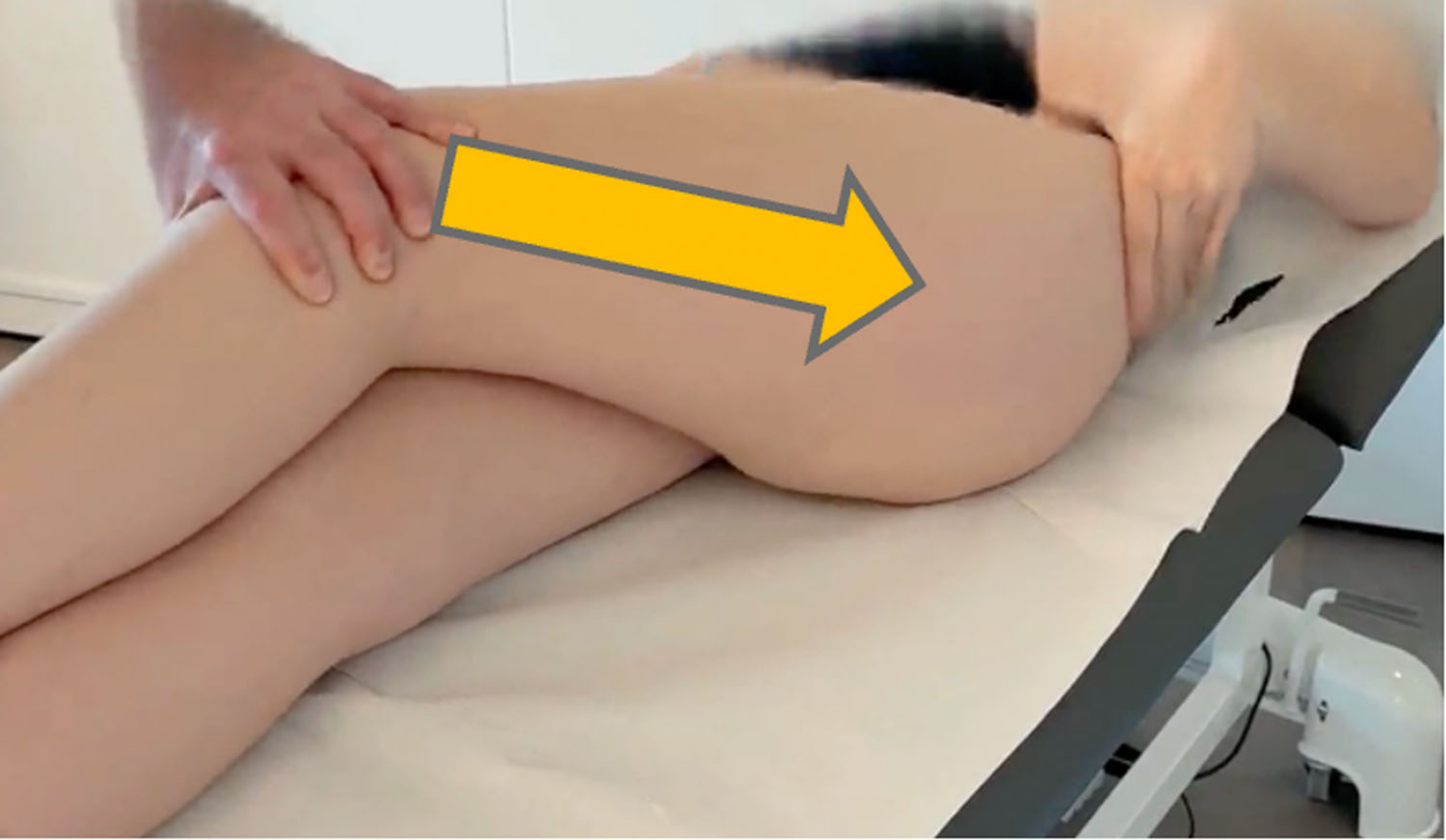
Lateral hip instability test: axial pressure on the adducted thigh with patient showing deep supratrochanteric hip pain.

The lateral hip instability test is performed with the patient in supine position, with both legs straight on the table. The painful leg is passively adducted by the examiner and crossed over the contralateral leg in slight flexion, until the end of ROM (maximum adduction). With the leg in neutral or slight rotation, the examiner then applies one to five short pushes with increasing force in the long axis of the femur; in case of pain, no extra pushes are applied (Figure 1). The lateral hip instability test is positive if it results in deep supratrochanteric hip pain, reproducing the pain while standing or walking. The aforementioned pain results from lateral or posterolateral hip subluxation, which can occur during the test in unstable hips, due to the pressure exerted on the posterosuperior acetabular rim and labrum. If the lateral hip pain is superficial and reproducible with palpation, one must instead consider gluteal or fascia lata pathologies.

### Statistical analysis

An a priori sample size calculation indicated that 130 patients (65 per group) were needed to determine a difference of 3° in LCEA between painful hips that tested positive versus negative using the lateral hip instability test. We assumed a standard deviation of 6° [8, 17, 20] and an allocation ratio of 1:1 from the surgeon's previous clinical experience in non‐arthritic patients. The sample size was calculated using a statistical power of 80%, a two‐tailed *t* test and an alpha level of 0.05.

Descriptive statistics were used to summarise the data, including mean, standard deviation, and range for continuous variables, as well as proportions and percentages for categorical variables. Differences between painful versus asymptomatic hips, and between hips with a positive versus negative lateral hip instability test were assessed using the chi‐squared test or Fisher's exact test for categorical variables, and the *t* test (normally distributed) or Wilcoxon test (not normally distributed) for continuous variables. Normality of distributions was assessed with the Shapiro–Wilk test. The accuracy of the lateral hip instability test was assessed only for painful hips using sensitivity, specificity, positive and negative predictive values, and positive and negative likelihood ratios, by taking as a reference LCEA ≤ 20° for frank dysplasia, LCEA ≤ 25° for borderline dysplasia, and a combination of positive COS, PWS and ISS for acetabular retroversion, as well as presence of labral hypertrophy. Statistical analyses were performed using R Statistical Software (v4.4.1; R Core Team 2024) [27] and Epi package [4].

## RESULTS

Asymptomatic (*n* = 108) and painful (*n* = 159) hips had comparable age (35 ± 9 vs. 34 ± 9, *p* = 0.349) and sex (67% vs. 67%, *p* = 1.00) distributions, as well as comparable ROMs, apart from flexion (105 ± 5° vs. 102 ± 6°, *p* < 0.001) and internal rotation (21 ± 13° vs. 16 ± 13°, *p* < 0.001) (Table 1). Asymptomatic hips were significantly less likely to have a positive clinical test compared to painful hips. Furthermore, asymptomatic hips had lower proportions of Tönnis Grade 1 (13% vs. 24%, *p* = 0.028), but had comparable Tönnis angles (5 ± 6° vs. 6 ± 7°, *p* = 0.568), LCEAs (27 ± 7° vs. 26 ± 8°, *p* = 0.449) and proportions of hips with acetabular dysplasia (17% vs 25%, *p* = 0.131) or retroversion (32% vs. 38%, *p* = 0.435).

Of the 159 painful hips, 69 (43%) had a positive lateral hip instability test (Table 2). Compared to the hips which tested negative, the hips which tested positive were younger (33 ± 9 vs. 36 ± 9, *p* = 0.028), more frequently female (81% vs. 57%, *p* = 0.001), more frequently painful while walking (97% vs. 74%, *p* < 0.001), had greater internal rotation (19 ± 15° vs. 13 ± 12°, *p* = 0.031) and abduction (47 ± 8° vs. 42 ± 7°, *p* < 0.001). The hips which tested positive had a greater proportion of Tönnis Grade 0 (84% vs. 70%; *p* = 0.042), a higher Tönnis angle (9 ± 8° vs. 3 ± 5°, *p* < 0.001), a lower LCEA (22 ± 8° vs. 29 ± 6°, *p* < 0.001), as well as greater proportions of frank (49% vs. 6%, *p* < 0.001) and borderline (70% vs. 24%, *p* < 0.001) dysplasia. They also had greater proportions of positive PWS (68% vs. 39%, *p* < 0.001), positive ISS (54% vs. 30%, *p* = 0.003), as well as the combination of positive COS, PWS and ISS (52% vs. 27%, *p* = 0.002), which indicates acetabular retroversion.

**Table 2:** Comparative statistics for painful and asymptomatic hips with negative versus positive lateral hip instability test.

	Painful hips							Asymptomatic hips							
	Negative lateral hip instability test (*n* = 90)			Positive lateral hip instability test (*n* = 69)				Negative lateral hip instability test (*n* = 104)				Positive lateral hip instability test (*n* = 4)			
	Mean	±SD	Range	Mean	±SD	Range	*p* value	Mean	±SD	Range		Mean	±SD	Range	*p* value
	*n*	%		*n*	%			*n*	%			*n*	%		
Patient characteristics															
Age	35.7	±8.8	18.1–49.6	32.5	±8.8	18.0–49.6	**0.028**	35.2	±8.6	18.0–49.9		40.7	±9.5	27.6–49.6	0.219
Sex: female	51	57%		56	81%		**0.001**	69	66%			3	75%		1.000
Painful hip: left	49	54%		28	41%		0.109								
Duration of hip pain (months)	28.9	±39.5	0.5–240.0	34.2	±38.1	2.0–240.0	0.057								
Hip pain while sitting	58	64%		43	62%		0.868								
Hip pain while walking	67	74%		67	97%		**<0.001**								
ROM (°)															
Flexion	101.2	±5.7	90.0–120.0	102.1	±5.5	95.0–115.0	0.229	104.6	±5.1	95.0–120.0		106.3	±9.5	100.0–120.0	0.883
Extension	9.8	±1.3	0.0–10.0	10.0	±0.0	10.0–10.0	0.129	10.0	±0.5	5.0–10.0		10.0	±0.0	10.0–10.0	1.000
External rotation	39.2	±11.0	15.0–70.0	37.7	±9.6	20.0–70.0	0.527	36.9	±9.9	20.0–70.0		40.0	±0.0	40.0–40.0	0.337
Internal rotation	13.4	±11.7	−15.0^a^ to 60.0	19.0	±15.1	0.0–60.0	**0.031**	20.4	±12.4	−10.0^b^ to 70.0		28.8	±17.0	15.0–50.0	0.322
Abduction	42.3	±7.2	20.0–60.0	47.0	±7.9	20.0–60.0	**<0.001**	44.6	±7.3	25.0–70.0		48.8	±2.5	45.0–50.0	0.129
Adduction	29.9	±0.7	25.0–30.0	29.7	±1.7	20.0–30.0	0.773	30.0	±0.0	30.0–30.0		30.0	±0.0	30.0–30.0	1.000
Clinical examination															
Painful gluteal muscles	16	18%		3	4%		**0.012**	1	1%			0	0%		1.000
Painful fascia lata	12	13%		4	6%		0.183	0	0%			0	0%		1.000
Positive FADIR	72	80%		68	99%		**<0.001**	9	9%			2	50%		0.051
Positive apprehension test	20	22%		26	38%		**0.036**	0	0%			0	0%		1.000
Radiographic evaluation															
Tönnis grade															
0	63	70%		58	84%		**0.042**	90	87%			4	100%		1.000
1	27	30%		11	16%			14	13%			0	0%		
Tönnis angle (°)	3.2	±5.3	−5.0 to 24.0	8.6	±7.7	−5.0 to 40.0	**<0.001**	4.5	±5.8	−5.0 to 21.0		13.0	±1.4	12.0–15.0	**0.006**
LCEA (°)	29.3	±5.9	3.0–42.0	22.1	±7.7	0.0–40.0	**<0.001**	27.3	±7.1	7.0–45.0		18.3	±1.3	17.0–20.0	**0.006**
LCEA ≤ 20°	5	6%		34	49%		**<0.001**	14	13%			4	100%		**0.001**
LCEA ≤ 25°	22	24%		48	70%		**<0.001**	39	38%			4	100%		**0.023**
Positive COS	72	80%		56	81%		1.000	77	74%			2	50%		0.292
Positive PWS	35	39%		47	68%		**<0.001**	48	46%			1	25%		0.625
Positive ISS	27	30%		37	54%		**0.003**	36	35%			1	25%		1.000
Positive: COS, PWS, ISS	24	27%		36	52%		**0.002**	34	33%			1	25%		1.000
Positive AIIS	58	64%		51	74%		0.230	60	58%			2	50%		1.000

*Note*: Bold values indicate a statistically significant difference.

Abbreviations: AIIS, anterior inferior iliac spine sign; COS, cross‐over sign; FADIR, Flexion Adduction Internal Rotation; ISS, ischial spine sign; LCEA, lateral centre‐edge angle; n, number; PWS, posterior wall sign; ROM, range of motion; SD, standard deviation.

^a^ One hip was blocked at 15° of external rotation (no internal rotation possible).

^b^ The contralateral hip was blocked at 10° of external rotation (no internal rotation possible).

Of the 108 asymptomatic hips, 4 (4%) had a positive lateral hip instability test (Table 2). Compared to the hips which tested negative, the hips which tested positive tended to have greater proportion of positive FADIR test (50% vs. 9%, *p* = 0.051), and had a higher Tönnis angle (13 ± 1° vs. 5 ± 6°, *p* = 0.006), lower LCEA (18 ± 1° vs. 27 ± 7°, *p* = 0.006), as well as greater proportions of frank (100% vs. 13%, *p* = 0.001) and borderline (100% vs. 38%, *p* = 0.023) dysplasia.

In painful hips, the lateral hip instability test had a sensitivity of 63% and a specificity of 96% for detecting borderline dysplasia (LCEA ≤ 25°) and/or acetabular retroversion (positive COS, PWS and ISS), which are radiographic signs of lateral or posterolateral undercoverage (Table 3). Furthermore, the positive likelihood ratio was 16.3, and the negative likelihood ratio was 0.39. As for detecting frank dysplasia (LCEA ≤ 20°) and/or acetabular retroversion (positive COS, PWS and ISS) in painful hips, the lateral hip instability test had a sensitivity of 68% and a specificity of 87%, as well as a positive likelihood ratio of 5.4 and a negative likelihood ratio of 0.36.

**Table 3:** Sensitivity, specificity, predictive values and likelihood ratios of lateral hip instability test in painful hips, using LCEA as standard reference for acetabular dysplasia and combination of positive COS, PWS and ISS as standard reference for acetabular retroversion.

	TP	FP	FN	TN	Sensitivity		Specificity		PPV	NPV	LR(+)	LR(−)
					Estimate (95% CI)		Estimate (95% CI)		Estimate (95% CI)	Estimate (95% CI)	Estimate (95% CI)	Estimate (95% CI)
Acetabular dysplasia ≤ 20°	34	35	5	85	0.87 (0.73–0.96)		0.71 (0.62–0.79)		0.49 (0.37–0.62)	0.94 (0.88–0.98)	2.99 (2.21–4.05)	0.18 (0.08–0.41)
Acetabular dysplasia ≤ 25°	48	21	22	68	0.69 (0.56–0.79)		0.76 (0.66–0.85)		0.70 (0.57–0.80)	0.76 (0.65–0.84)	2.91 (1.94–4.36)	0.41 (0.29–0.59)
Acetabular retroversion (3 signs)	36	33	24	66	0.60 (0.47–0.72)		0.67 (0.56–0.76)		0.52 (0.40–0.64)	0.73 (0.63–0.82)	1.80 (1.27–2.55)	0.60 (0.43–0.84)
Acetabular dysplasia ≤ 20° or retroversion (3 signs)	60	9	28	62	0.68 (0.57–0.78)		0.87 (0.77–0.94)		0.87 (0.77–0.94)	0.69 (0.58–0.78)	5.38 (2.87–10.07)	0.36 (0.27–0.50)
Acetabular dysplasia ≤ 25° or retroversion (3 signs)	67	2	40	50	0.63 (0.53–0.72)		0.96 (0.87–1.00)		0.97 (0.90–1.00)	0.56 (0.45–0.66)	16.28 (4.15–63.87)	0.39 (0.30–0.50)

Abbreviations: 95% CI, 95% confidence interval; COS, cross‐over sign; FN, false negative; FP, false positive; ISS, ischial spine sign; LCEA, lateral centre‐edge angle; NPV, negative predictive value; PPV, positive predictive value; PWS, posterior wall sign; TN, true negative; TP, true positive.

One hundred fourteen of the 159 patients had hip MRA. Subgroup analysis showed that 83 of the 114 (73%) had labral hypertrophy visible on MRA, which is considered as a sign of hip instability. Seventy‐eight (68%) patients had posterolateral labral hypertrophy, while five (4%) had lateral. Of the 83 patients with labral hypertrophy, 58 presented with positive lateral hip instability test (Table 4). The lateral hip instability test had a sensitivity of 70% and a specificity of 97% for detecting labral hypertrophy. Furthermore, the positive likelihood ratio was 21.7, and the negative likelihood ratio was 0.31.

**Table 4:** Subgroup analysis of patients who had hip MRA, comparing labral hyperplasia to the results of the lateral instability test.

		Lateral instability test	
		Positive	Negative
MRA	Positive (labral hyperplasia)	58	25
	Negative (no labral hyperplasia)	1	30

Abbreviation: MRA, magnetic resonance arthrogram.

## DISCUSSION

Diagnosis of hip instability is a relevant clinical problem, as unstable hips may warrant more complex and earlier surgical treatments than stable hips. The present study has found that, in painful hips, the lateral hip instability test had a sensitivity of 63%, a specificity of 96%, a positive likelihood ratio of 16.3 and a negative likelihood ratio of 0.39 for detecting borderline dysplasia and/or acetabular retroversion, which are aetiologies leading to lateral or posterolateral hip instability [16, 44]. In comparison, the lateral hip instability test had a sensitivity of 68%, a specificity of 87%, a positive likelihood ratio of 5.4 and a negative likelihood ratio of 0.36 for detecting frank dysplasia and/or acetabular retroversion. Therefore, if the lateral hip instability test is positive, clinicians can be quite certain that a patient has hip instability resulting from lateral and/or posterolateral undercoverage of the femoral head.

The lateral hip instability test could be performed for every patient presenting with hip pain. A positive test is a sign of clinical hip instability. In combination with radiological signs of acetabular dysplasia and/or retroversion, a positive test can confirm the diagnosis of hip pain due to lateral or posterolateral femoral head undercoverage. However, femoral head undercoverage can be asymptomatic and/or not result in hip instability, in which cases, surgical treatment may not be necessary. Hence, a combination of (i) presence of hip pain, (ii) a positive lateral hip instability test and (iii) radiological signs of acetabular dysplasia and/or retroversion would demonstrate hip instability and indicate the need for surgical treatment. The exact choice of surgical treatment is at the discretion of the surgeon and would depend on patient characteristics.

In painful hips, the lateral hip instability test showed higher specificity for borderline dysplasia and/or acetabular retroversion compared to frank dysplasia and/or acetabular retroversion (96% vs. 87%). Since borderline dysplasia is harder to identify clinically compared to frank dysplasia, a lower sensitivity would be expected for borderline dysplasia. Given that specificity and sensitivity are inversely related, specificity increases as sensitivity decreases [21]. The high number of false negatives of the lateral hip instability test may be due to patients experiencing hip pain caused by impingement instead of instability, or due to anterior undercoverage of the femoral head, which is common in dysplastic hips [16, 22, 44]. In both cases, we would expect the lateral hip instability test to be negative, even if the patient has radiographically confirmed dysplasia and/or retroversion.

Interestingly, there were no significant differences in radiographic signs and angles between painful versus asymptomatic hips (Table 1). In most cases, painful hips in the present study had contralateral asymptomatic hips that were included as a control group. Since symmetry in hip morphology across sides has been reported in a number of studies [20, 48], patients may have bilateral dysplasia or retroversion that is only or mostly symptomatic on one side [11].

The three‐dimensional (3D) patterns of acetabular deficiencies in adult patients with Tönnis Grade 0 to 1 and acetabular dysplasia were originally described by Nepple et al. [22], who emphasised the frequent concomitance of acetabular retroversion with dysplasia and the need to not only diagnose the previously recognised anterosuperior acetabular deficiency. They distinguished (i) anterosuperior, (ii) global and (iii) posterosuperior acetabular deficiencies, corresponding to, respectively, (i) lateral and anterior undercoverage of the femoral head, (ii) lateral undercoverage with variable (but similar) degrees of anterior and posterior undercoverage, and (iii) lateral and posterior undercoverage. Furthermore, the American Association of Hip and Knee Surgeons symposium on hip dysplasia and structural instability [44] proposed a comprehensive classification for acetabular dysplasia based on the location of undercoverage of the femoral head. Posterior instability corresponds to posterior undercoverage, which could be due to retroversion and can exist even in a setting of normal LCEA; it would result in positive posterior apprehension test and positive PWS, ISS and COS, as well as decreased posterior wall index. Lateral/global instability corresponds to lateral undercoverage with variable degrees of anterior and posterior undercoverage, and would result in reduced LCEA, as well as the features of global instability and subluxation. Anterior instability corresponds to anterior undercoverage or excessive acetabular anteversion; it would result in positive anterior apprehension test and ‘prone apprehension‐relocation’ test, as well as normal LCEA, negative COS and PWS, and decreased anterior wall index. These two recent studies [22, 44] highlight the need to widen clinical reasoning beyond the search for anterior undercoverage, and to express hip instability due to bony deficiencies in 3 dimensions. Hence, dedicated clinical tests and radiographic signs are needed to diagnose hip instability originating from different patterns of undercoverage of the femoral head.

There are multiple clinical tests that can be used to differentiate between stable and unstable painful hips. Since anterior hip instability is the most common type of instability in the adult population, there are many clinical tests to diagnose it [7, 10, 13, 30, 33, 39, 42, 45]. Among these tests, Abduction HyperExtension External Rotation (AB‐HEER) has been shown to have excellent sensitivity (80.6%) and specificity (89.4%) [10, 31]. In contrast, only one clinical test is widely used to diagnose posterior hip instability: the posterior apprehension test [6, 45]. Furthermore, we have not identified any clinical test in the published literature for the diagnosis of lateral hip instability due to lateral or posterolateral undercoverage of the femoral head. In dysplastic hips specifically, the centre of the femoral head has been shown to displace laterally and cranially during walking [14, 35]; thus, it seemed necessary to develop a clinical test that reproduces this subluxation.

There are some limitations associated with this retrospective study. First, we used radiographic signs of acetabular dysplasia and/or retroversion as an equivalent for lateral and/or posterolateral hip instability. Hip instability may only be visualised with fluoroscopy or arthroscopy during clinical manoeuvres; however, this would be difficult for both ethical and organisational reasons. Second, since this was a preliminary study describing and evaluating the lateral hip instability test for the first time, there was only one surgeon (the test creator) performing the test on all the patients. Future studies are needed to confirm the test's reproducibility across different clinicians and centres, as well as to evaluate the intra‐rater reliability. Third, computed tomography (CT) could have been useful to confirm undercoverage of the femoral head and its location by assessing the 3D hip anatomy; however, CT is not systematically performed in our clinical practice for patients with hip pain. Fourth, only patients who required and consented to surgery had a hip MRA, which was used to assess labral hypertrophy, that is associated with hip instability [2, 6, 15, 23, 24, 25, 34]. Thus, we decided to perform a subgroup analysis on 114 out of 159 patients who had MRA. Fifth, radiographic criteria for hip instability are currently not clearly defined, thus the choice of a reference standard for assessing diagnostic accuracy was subjective. In the recent consensus on hip pain in young and middle‐aged active adults [30], the following three radiographic parameters were recommended for diagnosis of hip instability: iliofemoral line, iliocapsularis‐to‐rectus femoris ratio and Shenton line. Other new radiographic signs for hip instability include the upsloping sourcil sign [46], the Gothic arch angle sign [49] and the Femoro‐Epiphyseal Acetabular Roof (FEAR) index [5, 47]; however, due to their novelty, none of these signs are considered as the gold standard for diagnosis of hip instability yet. We decided to use common radiographic signs for acetabular dysplasia and retroversion, since they are a part of standard clinical practice, and they could facilitate future comparisons with other studies. Finally, it is possible that due to the small sample size of asymptomatic hips with positive lateral hip instability test (*n* = 4), the comparison against asymptomatic hips with negative test has a high risk of Type II error: there may be differences between groups, but these do not appear as significant.

## CONCLUSION

The lateral hip instability test is a new clinical test that can discriminate between stable versus unstable painful hips with lateral and/or posterolateral undercoverage of the femoral head. It has a sensitivity of 63%, a specificity of 96%, a positive likelihood ratio of 16.3 and a negative likelihood ratio of 0.39 for detecting borderline dysplasia and/or acetabular retroversion. Therefore, if the lateral hip instability test is positive, clinicians can be quite certain that a patient has hip instability resulting from lateral and/or posterolateral undercoverage.

## AUTHOR CONTRIBUTIONS

Study design, data collection, data interpretation, manuscript editing and final approval: Michael Wettstein. Study design, data analysis, data interpretation, manuscript writing and final approval: Kinga Michalewska. Study design, data analysis, data interpretation, manuscript writing and final approval: Sonia Ramos‐Pascual. Data collection, manuscript editing and final approval: Stefan Hefer. Data collection, manuscript editing and final approval: Elyazid Mouhsine. Study design, data interpretation, manuscript editing and final approval: Mo Saffarini.

## CONFLICT OF INTEREST STATEMENT

The authors declare no conflicts of interest.

## ETHICS STATEMENT

All patients provided written informed consent for use of their anonymised data.

## Data Availability

Upon reasonable request, the authors can provide access to the data used for all analyses and analytic code.
